# Systematic Review: Impact of Interferon-based Therapy on HCV-related Hepatocellular Carcinoma

**DOI:** 10.1038/srep09954

**Published:** 2015-05-12

**Authors:** Ching-Sheng Hsu, You-Chen Chao, Hans Hsienhong Lin, Ding-Shinn Chen, Jia-Horng Kao

**Affiliations:** 1 Division of Gastroenterology, Department of Internal Medicine, Taipei Tzu Chi Hospital, Buddhist Tzu Chi Medical Foundation, Taipei, Taiwan; 2 School of Medicine, Tzu Chi University, Hualien, Taiwan; 3 Graduate Institute of Clinical Medicine, National Taiwan University College of Medicine, Taipei, Taiwan; 4 Department of Internal Medicine, National Taiwan University Hospital, Taipei, Taiwan; 5 Genomics Research Center, Academia Sinica, Nankang, Taiwan; 6 Department of Medical Research, National Taiwan University Hospital, Taipei, Taiwan; 7 Hepatitis Research Center, National Taiwan University Hospital, Taipei, Taiwan

## Abstract

Hepatitis C virus (HCV) is the leading cause of hepatocellular carcinoma (HCC), and several antiviral agents are available for the treatment of chronic HCV infection. However, the impact of antiviral therapy on the long-term outcomes of HCV-related HCC patients remains inconclusive. We aimed to examine the impact of antiviral therapy on the long-term outcomes of HCV-related HCC patients. We conducted a systematic review using PRISMA guidelines to identify trials and English-language literature from PubMed, Ovid MEDLINE, Scopus and the Cochrane Library database till August 2014. Randomized trials of antiviral treatments examining the effects of antiviral therapy on CHC patients and HCV-related HCC patients were screened and selected. We identified 6 trials evaluated the effectiveness of interferon (IFN)-alfa treatment, 3 studies examined pegylated interferon-alfa treatment, and 2 studies examined IFN-beta treatment. IFN-based therapy may decrease HCC incidence in HCV cirrhotic patients after a >5-year follow-up, improve liver reserve, decrease HCC recurrence rate, and increase survival rate in HCV-related HCC patients after curative HCC therapy. In conclusion, IFN-based therapy is beneficial and may be recommended in the management of HCV-related HCC patients who are IFN eligible.

Hepatitis C virus (HCV) infection is the leading cause of chronic liver diseases, leading to the progression of liver disease from chronic hepatitis to cirrhosis, with the complications of liver failure and hepatocellular carcinoma (HCC)^1^,^2^. Therefore, eradication of HCV viremia has been recognized as a reasonable way to delay or halt disease progression, hepatic complications and HCC of chronic hepatitis C CHC) patients. Before 2011, interferon (IFN)-based therapy, including dual therapy with pegylated interferon (PEG-IFN) plus ribavirin, was the standard of care of HCV patients in most part of the world. Since 2011, several effective direct antiviral agents (DAAs) have been proven by the U.S. Food and Drug Administration for the treatment of CHC. Although previous studies examined the effectiveness of anti-HCV therapy on disease progression, long-term complications and HCC risk of CHC patients, most of them included non-HCC, highly selected subjects and did not adequately control confounders^3^. Moreover, even fewer studies focused on PEG-IFN based treatment^4^,^5^, and the impact of DAAs on the long-term complications and HCC risk of CHC patients remain largely unknown^6^.

Because understanding the impact of antiviral regimens on HCV-related HCC is essential for the treatment plan designing of CHC patients with or without HCC, several trials have examined the impact of antiviral agents on HCV-related HCC patients. However, most of these trials differ significantly in the study population, protocols, antiviral regimens, and definitions of outcomes^7^,^8^,^9^,^10^,^11^,^12^, whether antiviral regimens will be beneficial for HCV-related HCC patients are inconclusive and deserve further studies.

To this end, we conducted this systematic review to examine the impact of antiviral therapy on the long-term outcomes of HCV-related HCC patients. As the effect of antiviral therapy on the HCC risk of CHC patients remain unconcluded, and antiviral therapy is not likely to affect the long-term outcomes of HCV-related HCC patients when it does not decrease the HCC risk in CHC patients. We investigated the impact of antiviral regimens on the HCC incidence of patients with HCV infection first. Once positive findings were shown in the first part, we then examined the long-term outcomes of HCV-related HCC patients by searching relative references and examining the benefits as well as harms of antiviral agents for HCV-related HCC patients.

## Methods

### Scope

A review protocol and an analytic framework were developed with input and discussion from the authors to set clear questions, ensure a standardized and repeatable review process between reviewers (Supplementary Figure and Supplementary Protocol), and included the following key questions:

1. What is the effect of antiviral treatment on the HCC incidence of patients with chronic HCV infection?
2. What is the effect of antiviral treatment on HCC outcomes of patients with HCV-related HCC (including the recurrence or progression of HCC)?
3. What is the effect of antiviral treatment on clinical outcomes (including the survival and long-term complications) of patients with HCV-related HCC?
4. What are the comparative harms associated with antiviral treatments in patients with HCV-related HCC?

### Data Sources and Searches

We followed the PRISMA recommendations for systematic literature analysis, which is an evidence-based set of items for reporting in systematic reviews and meta-analyses, and focuses on ways to ensure the transparent and complete reporting^13^. We performed computer–based searches of the PubMed, Ovid MEDLINE, Scopus, and the Cochrane Library database for clinical trials in original articles between 1947 and August 2014. We searched using combinations of the following terms: “interferon” or “antiviral therapy” or “pegylated interferon” or “ribavirin” or “DAA” or “hepatitis C” or “chronic hepatitis C” or “HCV” and “hepatocellular carcinoma” or “HCC” in the title or abstract fields. We screened each abstract resulting from these searches for eligibility. We also examined reference lists of each selected original article that might meet our eligibility requirements.

### Study Selection

Two reviewers independently evaluated studies for inclusion. We included randomized trials on HCV-related HCC patients that investigated IFN-based therapy, including mono-therapy with IFN or PEG-IFN alfa; dual therapy with IFN plus ribavirin or PEG-IFN alfa plus ribavirin; and triple therapy with PEG-IFN (alfa−2a or −2b), ribavirin, and either telaprevir or boceprevir or newer DAAs. Clinical outcomes were mortality, cirrhosis, hepatic decompensation, hepatocellular carcinoma, and need for transplantation. HCC outcomes were the recurrence and progression of HCC. Sustained virological response (SVR) was defined as the absence of detectable HCV RNA in the serum 6 months after the end of a course of therapy. Biological response (BR) was defined as a decrease in serum ALT to within the normal range after treatment. Harms included withdrawals due to adverse events, serious adverse events, neutropenia, anemia, psychological adverse events, influenza-like symptoms, and rash.

We restricted the inclusion to English-language articles. Article selection was determined a priori using the following inclusion criteria: (i) only adults were included in the trial; (ii) patients with chronic HCV infection were included in the study examining effects of antiviral treatments on HCC incidence; and patients with HCV-related HCC were included in the study examining effects of antiviral treatments on HCC outcomes; (iii) the study used IFN- or PEG-IFN-based therapy combined with or without other antiviral agents. We excluded: (i) review articles, (ii) editorials; (iii) letters to the editor (iv) case reports; (v) research protocols; (vi) duplicated records; (vii) sub-analyses of the same clinical trial; if the final study results had been published; (ix) interim reports if the final results had been published; (x) regimens with antiviral drugs not approved by the U.S. Food and Drug Administration for HCV infection.

### Data Abstraction

One investigator abstracted details of the study, and a second investigator reviewed data for accuracy. The methodological quality of each study was assessed using the Cochrane Collaboration’s tool for assessing risk of bias in randomized trials (Table 1)^14^. Two investigators independently reviewed all studies and assigned a value of “low risk,” “high risk,” or “unclear” to the following: a) random sequence generation, b) allocation concealment, c) blinding of participants and personnel, d) adequate assessment of each outcome, e) selective outcome reporting avoided, and f) Other bias (reports for adverse effects of intervention). Discrepancies were resolved through consensus. The following data were extracted from each report: publication type (original article or conference abstract); number of patients; antiviral regimens; status of liver cirrhosis or liver fibrosis stage; percentage of patients with hepatitis B or hepatitis C; period of follow-up; incidence of HCC; recurrence rate of HCC; overall survival (OS); and adverse effects of antiviral agents.

## Results

**Figure 1:**
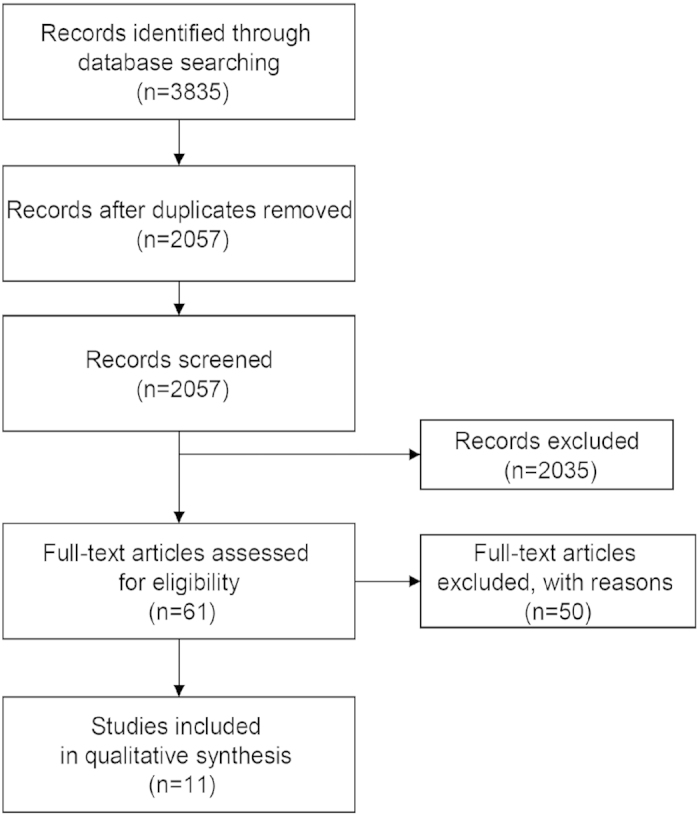
Summary of evidence search and selection.

The flow chart of the systematic review is shown in Fig. 1. Our initial search yielded 3835 articles. Ultimately, 11 randomized controlled trials (RCTs) were chosen for review, including 5 investigated antiviral treatments and HCC incidence in CHC patients, and 6 examined antiviral treatments and HCC outcomes in HCV-related HCC patients who had received curative therapy for HCC. Six trials evaluated effectiveness of IFN-alfa based treatment, 3 examined the effect of pegylated interferon-alfa, and 2 studies examined the effect of IFN-beta treatment. No RCT ever investigated the effects of antiviral treatments on HCV-related HCC patients without curative HCC therapy, and none evaluated the effectiveness of IFN plus ribavirin or DAAs.

### The impact of antiviral treatments on the incidence of HCC in patients with HCV infection

Five RCTs, including 1926 patients, examining the impact of antiviral agents on patients with HCV infection were identified, and summarized in Table 2^4^,^5^,^15^,^16^,^17^,^18^. Four trials included compensated cirrhotic patients, one trial (HALT-C) included patients with cirrhosis or advanced fibrosis (Ishak fibrosis scores ≥3)^4^, and 2 trials included subjects who failed previous antiviral treatment (HALT-C & EPIC3)^4^,^5^. One study used IFN-beta^16^, 2 used IFN-alfa^17^,^18^, 1 used PEG-IFN alfa−2a (HALT-C)^5^ and 1 used PEG-IFN alfa−2b (EPIC3) for the treatment of CHC patients^5^.

Four studies reported the improvement of serum alanine aminotransferase (ALT) levels, and decreased serum HCV RNA level in treated subjects. A biochemical end-of–therapy response was observed in 5/38 (13%) IFN-beta treated patients [2/23 (9%) in controls], and 6/47 (12.8%) IFN-alfa treated patients [0/39 in controls]^16^,^17^. Although Nishiguchi *et al.* did not identify a significant difference in the normalization of ALT level between groups, they found that ALT deceased to <80 IU/L in 19/45(42.2%) IFN-alfa treated patients [9/45 (20%) in controls]. Moreover, they also found lower AFP and higher serum albumin levels in IFN-treated patients. For PEG-IFN-alfa 2a treatment, HALT-C study showed a 0.45–fold decline of serum ALT levels among treated patients at 1.5 years (0.21 times among control patients), a 0.47-fold decline among treated patients at 3.5 years (0.19 times among control patients), and a significant difference of 0.28 times the upper limit of the normal range (95% CI, 0.12 to 0.44; P < 0.001).

Two studies (Nishiguchi *et al.* & HALT-C)^4^,^18^ reported the improvement of liver histology in treated subjects, and 1 study (EPIC3)^5^ reported a longer time to disease progression (clinical events indicating disease progression, development of Child-Pugh class B, and new/increased varices) in patients receiving PEG-IFN alfa−2b compared with control patients (P = 0.007; HR, 1.564 [95% CI: 1.130–2.166]). A decreased incidence of HCC was observed in 2 trials with a follow-up period more than 5 years (Nishiguchi *et al.* & HALT-C)^15^,^18^, but not in trials with a follow-up less than 5 years^5^^,^^15^,^16^,^17^.

Therefore, IFN-treated CHC patients had a better liver function tests, liver histology, and slower liver disease progression than non-IFN-treated controls. Moreover, IFN-treated CHC cirrhotic patients had a lower HCC incidence than non-IFN-treated controls after a >5-year of follow-up.

### The impact of antiviral treatment on the clinical outcomes of patients with HCV-related HCC

Six RCTs were identified to examine the impact of antiviral agents for patients with HCV-related HCC after curative HCC therapy. A total of 374 HCV-related HCC patients, including 201 IFN-treated patients and 173 controls, are summarized in Table 3^7^,^8^,^9^,^10^,^11^,^12^. All trials included HCV-related HCC patients who received curative HCC therapy, including surgical resection in 3^8^,^10^,^11^, percutaneous ethanol injection(PEI) or resection in 1^9^, PEI in 1^7^ and transcatheter arterial chemoembolization(TACE) plus PEI in 1^12^. One study used IFN-beta^9^, 5 used IFN-alfa^7^,^8^,^10^,^12^, and one used PEG-IFN alfa−2b plus ribavirin^11^ for the treatment of HCV-related HCC patients after curative HCC treatment.

Four studies reported SVR rates in IFN-treated patients^7^,^8^,^10^,^12^, and improved liver function tests in IFN-treated HCV-related HCC patients^7^,^8^,^10^,^11^. Patients receiving IFN-alfa treatment had SVR rate of 7–50% and sustained biological response (SBR) rate of 7–43%^7^,^8^,^10^,^12^. Although IFN-beta did not affect the SVR and SBR rates in HCV-related HCC patients, serum transaminases tended to decrease at the 12th month in IFN-beta treated group^9^. Moreover, increased serum albumin level was observed in HCV-related HCC patients receiving PEG-IFN alfa-2b plus ribavirin therapy, especially in SVR patients^11^. Six studies reported the overall survival difference between groups, including 4 reported a better survival rate in IFN-treated group (P < 0.05)^7^,^8^,^11^,^12^, one reported a marginal higher survival rate in the IFN-treated group (IFN vs. control: 24.3% vs. 5.8%; P = 0.49)^10^, and one reported no mortality until the end of the observation period^9^. In the study of Shiratori *et al.*, patients receiving IFN-alfa treatment had survival rates of 98%, 82%, 68%, and 53% at 1, 3, 5, and 7 years, respectively; untreated patients had survival rates of 96%, 84%, 48%, and 23% at 1, 3, 5, and 7 years, respectively. In addition, survival rates in SVR patients were 86%, 78%, and 68% at 3, 5, and 7 years, respectively; whereas survival rates in non-SVR patients were 80%, 65%, and 47% at 3, 5, and 7 years, respectively^7^. In patients receiving PEG-IFN-alfa−2b plus ribavirin treatment^11^ cumulative survival rates among HCV-related HCC patients were 100% after 1 year and 90.2% after 3 years in the IFN-treated subjects compared to 96.0% and 61.2% in the non-treated controls^11^. Therefore, IFN-treated patients who had received curative HCC therapy, had a better biochemical response, virological response, liver function reserve and survival rate than non-IFN-treated controls.

### The impact of antiviral treatment on the recurrence of HCC after patients receiving curative HCC therapy

Six studies reported the recurrence rate of HCC in HCV-related HCC patients after curative HCC therapy^7^,^8^,^9^,^10^,^11^,^12^ (Table 3). Five studies reported a lower recurrence rate of HCC in HCV-related HCC patients receiving IFN-based treatment^7^,^8^,^9^,^10^^,^^12^,^19^,^20^, while the study investigating the impact of PEG-IFN-alfa-2b plus ribavirin treatment did not identify a significant difference in cumulative first recurrence rates of HCC^11^. Because tumor recurrence after resection of HCC can occur early (<2 years) or late (≧2 years) as metastases or de novo tumors, four studies also examined the recurrence rates in HCV-related HCC patients in the second year or later, and found that IFN-based treatment was associated with a lower late recurrence rate^7^,^9^,^10^,^12^. Therefore, IFN-treated patients who had received curative HCC therapy had a lower recurrence rate, especially a lower late recurrence rate of HCC than non-IFN-treated controls.

### Adverse events associated with antiviral treatments in patients with HCV-related HCC

There was no available data for a direct comparison between HCV infected patients and HCV-related HCC patients in the adverse events after antiviral therapy. However, five studies investigating IFN-based therapy reported the associated adverse events, and most were expected symptoms such as fever, chills, myalgia, headaches fatigue, anorexia, weight loss, irritability, concentration, difficulty, sleep disturbance, depression, severe malaise, emotional liability, thrombocytopenia, leukocytopenia, hyperthyroidism, and deterioration in Child’s score.

## Discussion

Antiviral regimens have been documented to result in decreasing risks of HCC and liver cirrhosis complication in CHC patients^3^. Although understanding the impact of antiviral agents on HCV-related HCC patients is critical for making treatment decision in such patients, whether antiviral regimens are beneficial for HCV-related HCC remain inconclusive. In this systematic review, we concluded that IFN-based treatment may decrease HCC incidence in HCV-related cirrhotic patients. Moreover, IFN-based therapy may improve liver reserve, significantly increase the long-term survival and decrease the HCC recurrence rate of HCV-related HCC patients after curative HCC therapy. Therefore, IFN-based therapy is beneficial, and may be recommended in the management of HCV-related HCC patients who are IFN eligible.

In lieu of direct evidence on long-term clinical outcomes of antiviral agents, SVR rate is the primary outcome measurement for the effectiveness of antiviral regimens^21^. However, most of the selected trials investigated the effects of IFN-alfa- or beta-based therapy that are known to have a lower efficacy than PEG-IFN or DAAs-based regimens^22^. Therefore, studies investigating IFN regimens usually have a modest virological and biochemical response after treatment, and a longer observational period is needed for significant differences been detected in the target population. That is why a significant decline of HCC incidence in CHC patients receiving IFN treatment was observed in studies with a period of follow-up more than 5 years^15^,^18^, but not in ones with a shorter period of follow-up. Interestingly, even the differences in terms of virological response and improvement in liver reserve are modest (Table 3), IFN-based therapy may significantly reduce HCC recurrence rate in HCV-related HCC patients after curative HCC therapy. This may be attributed to the additional inhibitory effects of IFN on liver carcinogenesis^23^,^24^.

Compared to untreated controls, 8 studies showed a biochemical response with serum ALT decline, and 2 showed a better liver reserve in CHC patients after receiving IFN-based therapy^11^,^18^. Although 2 studies showed a potential benefit of IFN-based treatments for improving liver histology (Nishiguchi *et al.* & HALT-C)^15^,^18^, and 1 study (EPIC3) demonstrated a decreasing incidence of liver cirrhosis complications, including ascites, encephalopathy, and varices bleeding, in CHC patients receiving IFN-based treatments^5^. There is no study investigating the effects of antiviral regimens on liver histology and complications of liver cirrhosis in HCV-related HCC patients. Moreover, although two large RCTs (HALT-C & EPIC3)^4^,^5^ have examined the impact of PEG-IFN treatment on CHC cirrhotic patients, most of the enrolled patients failed initial antiviral treatments and received half-dose regimens, the long-term outcomes after PEG-IFN-based therapy in treatment-naive HCV cirrhotic patients remain unclear. Therefore, future studies examining the impact of PEG-IFN-based regimens on long-term outcomes in HCV-related HCC patients and treatment-naive HCV cirrhotic patients are needed. In addition, this review is limited by the lack of information regarding DAA regimens. As DAAs have become the backbones of standard of care for HCV infection, their impact on HCC risk and long-term outcomes in CHC patients with and without HCC warrants future studies.

Our study had a few limitations. First, a positive “publication” bias that tends to publish research with a positive outcome more frequently may be introduced into this study^25^. Moreover, because some inclusion criteria were used to select studies, a few studies might be excluded and missed during the literature search. Second, there exists substantially clinical heterogeneity in the selected randomized controlled trials. For examples, kinds of IFN, duration and dose of antiviral agents, and race that may be different in the prevalence of IL28B major allele, and all of these may hamper the validity of the pooled estimate and affect the ability to perform a metaanalysis^26^. Therefore, we performed descriptive evaluations for each study rather than combined the data together. However, descriptive evaluations for each study have shown consistent results among studies. Third, the time-to-event outcomes are likely affected by censoring and the duration of follow-up that are different among individual trials^27^, and the unavailability of individual data may limit the analysis of the clinical outcomes in patients with HCV-related HCC. Last, based on the sub-group analyses of patients with compensated cirrhosis in several large clinical trials^28^,^29^,^30^, the up-to-date guidance for the treatment of HCV infection of the American Association for the Study of Liver Diseases and the Infectious Diseases Society of America does not recommend mono or dual therapy with IFN for the treatment patients with HCV-related cirrhosis, and has recommended the use of highly potent DAA oral combination regimens for HCV infected patients. However, the long-term effects of the use of DAAs, including its effect on HCC, remain unclear and need be further examined. Considering the additional anti-tumorigenic effect of IFN, IFN-based therapy might be recommended in HCV-related HCC patients who are IFN eligible. However, future comparative studies are needed to confirm this speculation.

## Conclusions

This systematic review supports the beneficial effects of IFN-based treatment for decreasing HCC incidence in HCV-related cirrhotic patients, and HCC recurrence rate in HCV-related HCC patients after curative HCC therapy. Moreover, IFN-based therapy is associated with the improvement of serum ALT levels and better liver reserve in HCV-related HCC patients after curative HCC therapy. However, future studies are required to clarify the impact of DAAs on HCC risk and cirrhotic complication in CHC patients and HCV-related HCC patients after curative HCC therapy.

## Additional Information

**How to cite this article**: Hsu, C.-S. *et al.* Systemic Review: Impact of Interferon-based Therapy on HCV-related Hepatocellular Carcinoma. *Sci. Rep.* **5**, 9954; doi: 10.1038/srep09954 (2015).

## Supplementary Material

Supplementary Information

## Figures and Tables

**Table 1:** Quality of assessment of each included studies.

**Trial name or First author**	**Random sequence generation (selection bias)**	**Allocation concealment (selection bias)**	**Blinding of participants and researchers (performance bias)**	**Blinding of outcome assessment (detection bias)**	**Incomplete outcome data (attrition bias)**	**Selective reporting (reporting bias)**	**Other bias (reports for kinds of adverse effects for intervention )**
Bernardinello E, *et al.*/TVVH Study Group.	Low	Unclear	High	High	Low	Low	Low
Valla DC, *et al.*	Low	Unclear	High	High	Low	Low	Unclear
Nishiguchi S, *et al.*	Low	Unclear	High	High	Low	Low	Low
Di Bisceglie AM, *et al.*/ HALT-C Trial Investigators	Low	Unclear	High	High	Low	Low	Low
Bruix J, *et al.*/EPIC3 Study Group	Low	Unclear	High	High	Low	Unclear	Low
Kubo S, *et al.*	Low	Low	High	High	Low	Low	Low
Ikeda K, *et al.*	Unclear	Unclear	High	High	Low	Low	Low
Mazzaferro V, *et al.*	Low	Low	High	High	Low	Low	Low
Shiratori Y, *et al.*	Low	Low	High	High	Low	Low	Low
Miyaguchi S, *et al.*	Unclear	Unclear	High	High	Low	Low	Low
Ishikawa T, *et al*	Unclear	Unclear	High	High	Low	Low	High

**NOTE**. “Low” is “low risk of bias”, that means if bias is present, is unlikely to alter the results.

“Unclear” is “unclear risk of bias”, that means a risk of bias that raises some doubt about the results.

“High risk” is “high risk of bias”, that means bias may alter the results seriously.

**Table 2:** Summary of antiviral treatments on the clinical outcomes and HCC incidence in patients with HCV infection.

**Trial name or First author**	**Antiviral regimens**	**Number of patients (Treat group vs. non-treat group)**	**Study population**	**Period of Follow-up**	**Biological response (BR), Treat group vs. non-treat group**	**Virological response (VR), Treat group vs. non-treat group**	**Incidence of HCC and liver cirrhosis complication**	**Endpoint**	**Conclusion**	**References (published year)**
Bernardinello E, *et al.*/TVVH Study Group.	beta-IFN 6 Million units(MU) tiw for 6 months followed by 3 MU tiw for 6 months	61 (38 vs. 23)	CHC patients with cirrhosis	5 years	Normalization of ALT: 5/38 (13%) vs. 2/23 (9%)	Negative of serum HCV-RNA 4/38 (11%) vs. 0/23	The cumulative probability of liver decompensation at 60 months was 24% in treated and 35% in untreated cases.HCC developed in 2 treated and in 1 untreated patient.No significant reduction of cirrhosis related clinical events in treated patients.	Liver decompensation (variceal bleeding, ascites or hepatic encephalopathy), and HCC incidence	beta-IFN therapy does not improve either in BR or VR or reduction of cirrhosis related clinical events in CHC cirrhotic patients.	16 (1999)
Valla DC, *et al.*	IFN-alpha 2b 3MU tiw for 48 weeks	99 (47 vs. 52)	CHC patients with biopsy-prove compensated cirrhosis	160+/− 57 weeks	End-of-treatment (EOT)-BR was not observed in any control but in 6/47 treated patients (P < 0.02); SBR in 2 treated patients.	SVR in 2 treated patients	HCC developed in 9 controls and 5 treated patients; decompensation of cirrhosis occurred in 5 controls and 7 treated patients. Seven controls and 10 treated patients died.	Decompensation of cirrhosis, death, and HCC incidence.	A 48-week course of IFN therapy can induce EOT-BR, but fail to achieve sustained response and improve the 3-year outcome in patients with compensated HCV-related cirrhosis.	17 (1999)
Nishiguchi S, *et al.*	IFN-alpha 6 MU tiw for 12–24 weeks	90 (45 vs. 45)	CHC with compensated cirrhosis	2–7 years	ALT <80 IU/L: 19 vs. 9(P < 0.011). Mean ALT is comparable, but lower AFP, higher albumin, and improved histology in IFN group.	EOT-VR: 16 in IFN treated patients. HCV RNA disappeared: 7/45 (16%) vs. 0/45 (P = 0.018)	HCC was detected in 2 IFN-alpha patients and 17 controls. The risk ratio of IFN-alpha treatment versus symptomatic treatment was 0.067 (0.009–0.530; P = 0.010 Cox’s proportional hazards).	HCC incidence	IFN-alpha improved liver function and decreased incidence of HCC in CHC cirrhotic patients.	18 (1995)
Di Bisceglie AM, *et al.*/ HALT-C Trial Investigators	PEG-IFN alfa–2a 90 μg per week for 3.5 years.	1050 (517 vs. 533); 622 with non-cirrhotic fibrosis and 428 with cirrhosis	CHC patients (Ishak fibrosis scores ≥3) who did not have a SVR to initial PEG-IFN and ribavirin	3.5/ median 6.1(maximum, 8.7) yr	The level of serum aminotransferases, and histologic necroinflammatory scores all decreased significantly with treatment (P < 0.001).	The level of serum HCV RNA decreased significantly with treatment (P < 0.001)	After a 3.5 yr of follow-up, no significant difference between the groups in the rate of any primary outcome.After a 6.1 yr (median) of follow-up, HCC incidence: 37 of 515(7.2%) vs. 51/ 533 (9.6%; P = 0.24).After 7 years, the cumulative incidences of HCC in patients with cirrhosis: 7.8% vs. 24.2% (hazard ratio [HR], 0.45; 95% confidence interval [CI], 0.24–0.83). Treated patients with a ≥2-point decrease in the histologic activity index, based on a follow-up biopsy, had a lower incidence of HCC than those with unchanged or increased scores (2.9% vs. 9.4%; P = 0.03).	Death, HCC, hepatic decompensation, or an increase in the Ishak fibrosis score of 2 or more points.	Long-term therapy with PEG-IFN did not reduce disease progression rate in CHC patients with advanced fibrosis or cirrhosis, who failed to respond to initial treatment with PEG-IFN and ribavirin after a 3.5 yr of follow-up, but reduce the HCC risk in patients with cirrhosis who received PEG-IFN treatment after a 7 yr of follow-up.	4 (2008)
Bruix J, *et al.*/EPIC3 Study Group	PEG-IFN alfa−2b 0.5 μg/kg/week	626(311 vs. 315)	CHC compensated cirrhosis without HCC and had failed to respond to IFN alfa plus ribavirin	5 yr	Not available (NA)	NA	The time to disease progression was significantly longer for patients received PEG-IFN alfa-2b compared with controls (HR, 1.564; 95% CI: 1.130–2.166).Variceal bleeding was reported in 10 controls and 1 treated patient.No significant difference in time to first clinical event or decrease in the development of HCC with therapy.	Liver decompensation (variceal bleeding, Child-Pugh class C, ≧grade 2 hepatic encephalopathy, ascites requiring therapeutic paracentesis, and/or additional therapy), HCC, death, or liver transplantation	Maintenance therapy with PEG-IFN alfa-2b does not prevent HCC. There is a potential clinical benefit of long-term suppressive therapy in patients with preexisting portal hypertension.	9 (2011)

Five RCTs, including a total of 1,926 CHC patients with cirrhosis or advanced fibrosis, were identified to examine the impact of antiviral agents on patients with HCV infection. After a >2 years of follow-up, IFN-treated CHC patients had a better liver function tests, liver histology, and slower liver disease progression than non-IFN-treated controls. Moreover, IFN-treated CHC cirrhotic patients had a lower HCC incidence than non-IFN-treated controls after a >5-year of follow-up.

**Table 3:** Summary of antiviral treatment on the clinical outcomes and HCC recurrence after CHC patients receiving curative HCC therapy.

**Trial name or First author**	**antiviral regimens**	**Number of patients (Treat group vs. non-treat group)**	**Study population**	**Period of Follow-up (Treat group vs. non-treat group)**	**Biochemical and virological response, Treat group vs. non-treat group**	**Overall Survival (Treat group vs. non-treat group)**	**Incidence of HCC and liver cirrhosis complication**	**Endpoint**	**Comments**	**References (published year)**
Kubo S, *et al.*	IFN-alpha 6 MU daily for 2 weeks, then 3 times weekly for 14 weeks, and finally 2 weekly for 88 weeks	30 men (15 vs. 15)	HCV-related HCC after resection	Median follow-up period: 1817 days vs. 1487 days	In the IFN-alfa group, 2 SVR, 6 BR, and 7 non-responder. In the controls, postoperative serum ALT activity was greater than the reference range, and serum HCV RNA was detected during the follow-up.	The cumulative survival rate was higher in the IFN treated group than in the controls (P = 0.041).	Recurrent tumors were detected in 5 IFN-alpha treated patients and 12 control patients. The recurrence rate was significantly lower in the IFN-alpha group than in the control group (P = 0.037).	HCC recurrence rates and survival rate after resection.	Postoperative IFN-alpha therapy decreases HCC recurrence and improves the outcomes after resection of HCV-related HCC.	8 (2001) 20(2002)
Ikeda K, *et al.*	Natural IFN-beta 6MU twice a week for 36 months	20(10 vs. 10)	HCV cirrhotic patients after treatment with surgery or PEI	Median observation period: 25 months.	No patient lost HCV-RNA in the study. No significant differences in liver function test, but transaminases tended to decrease at the 12^th^ month in IFN group.	None died until the end of the observation period.	During follow-up, 7/10 (70.0%) patients in the controls, but 1/10 (10.0%) patients with IFN therapy had tumor recurrence. Cumulative HCC recurrence rates of the treated and untreated groups were 0% and 62.5% at the end of the first year, and 0% and 100% at the second year, respectively (log-rank test, P = 0.0004).	Cumulative HCC recurrence rates	Intermittent administration of IFN suppressed tumor recurrence after treatment with surgery or ethanol injection in patients with HCV-related chronic liver disease.	9 (2000)
Mazzaferro V, *et al.*	IFN-alpha 3 MU 3 times every week for 48 weeks.	150(76 vs. 74): 80 patients with HCV infection and 70 with mixed HCV + HBV infection	CHC patients undergoing resection for early- to intermediate-stage HCC	Median follow-up: 45 months	SVR in 2 patients (7%), 1 relapsed, while BR was observed in 2 additional patients.	Overall survival was 58.5%, and no significant difference in recurrence-free survival (RFS) (24.3% vs. 5.8%; P = 0.49).	A significant benefit on late recurrences (28 events) in HCV-pure patients adherent to treatment (HR: 0.3; 95% CI: 0.09–0.9; P = 0.04).	Primary end point was RFS; secondary end points were disease-specific and overall survival.	IFN does not affect overall prevention of HCC recurrence after resection, but it may reduce late recurrence in HCV-pure patients receiving effective treatment.	10 (2006)
Shiratori Y, *et al.*	IFN-alfa 3 times weekly for 48 weeks	74(49 vs. 25)	CHC patients with compensated cirrhosis, ≦3 nodules of HCC, and low HCV RNA loads (≦2 × 10^6^ copies/mL) and had a complete ablation of lesions by PEI therapy	Mean follow-up period: ≥7 years.	21 showed a SBR and 14 showed a SVR.	Patients treated with IFN had a survival rate of 68% at 5 years and 53% at 7 years; untreated patients had a survival rate of 48% at 5 years and 23% at 7 years.	The first recurrence of HCC was similar, but the rates of 2nd or 3rd recurrence were lower in the IFN group than in the untreated group.	The occurrence of new foci of HCC and survival rate	After tumor ablation by ethanol injection, IFN therapy may enhance patient survival in selected patients with CHC	7 (2003)
Miyaguchi S, *et al.*	Recombinant IFN-alfa 2b 3 MU 3 times a week for 4 months	46(22 vs. 24)	HCV related HCC patients with low HCV-RNA after being treated by TACE and PEI therapy	31.6 ± 10.6 months vs. 24.3 ± 7.1 months	11/22 (50%) had SVR in the treated group. 2/24(8.3%) un-treated patients had HCV-RNA spontaneous clearance	Survival rate in the IFN-treated group was higher than that in the untreated group (P = 0.01).	The incidence of secondary HCC was lower in the IFN-treated group than that in the untreated group.	Local recurrence and/or new development of primary tumor	IFN may be a therapy of choice in combination with TACE and PEI therapy for the treatment of HCC in low HCV-RNA patients.	12 (2002)
Ishikawa T, *et al*	PEG-IFN a-2b 1.5μg/kg body weight per week/RBV daily(< 60 kg: 600 mg, 60–80 kg: 800 mg, >80 kg: 1000 mg)	54 (29 vs. 25)	HCV-associated Stage I/II HCC after curative HCC treatment.	45 months	Serum albumin level decreased temporarily but subsequently increased, and improved hepatic functional reserve was observed in PEG-IFN a-2b/RBV therapy.	Cumulative survival rates were 100% after 1 year and 90.2% after 3 years in the IFN group compared to 96.0% and 61.2%, respectively, in the non-IFN group.	No significant differences in cumulative first recurrence rates	Survival rate, metachronous recurrence and hepatic functional reserve	PEG-IFN a-2b/RBV therapy after HCC treatment can improve hepatic functional reserve.	11 (2012)

**NOTE**. Six RCTs, including a total of 374 HCV-related HCC patients (201 IFN-treated patients and 173 controls), were identified to examine the impact of antiviral agents for patients with HCV-related HCC after curative HCC therapy. After a >25 months (median) of follow-up, CHC patients who had received curative HCC, IFN-treated patients had a better biochemical response, virological response, liver function reserve, survival rate, and a lower recurrence rate, especially a lower late recurrence rate of HCC, than non-IFN-treated controls.
