# Supplementary material for: Systematic Review: Impact of Interferon-based Therapy on HCV-related Hepatocellular Carcinoma
Source: Sci Rep. 2015 May 12;5:9954. doi: 10.1038/srep09954 (PMC4428066; doi:10.1038/srep09954)
Supplement: Supplementary Information [file srep09954-s1.pdf]

Title: Systemic Review: Impact of Interferon-based Therapy on HCV-related Hepatocellular Carcinoma

Ching-Sheng Hsu[1,2], You-Chen Chao[1,2], Hans Hsienhong Lin[1,2], Ding-Shinn Chen[3,4,5], Jia-Horng Kao*[3,4,6,7]

Supplementary 1.

Figure. Analytic framework for treatment of patients with HCV-related HCC.

This analytic framework outlines the population, interventions, and outcomes considered in the review. The population includes adults with chronic HCV infection and adults with HCV-related HCC. The interventions include interferon alfa-2a, interferon alfa-2b, pegylated interferon alfa-2a, pegylated interferon alfa-2b with or without ribavirin, or DAAs. Intermediate outcomes include biochemical and virological response (e.g. liver function, sustained virologic response, and histologic changes). Final HCC outcomes include the incidence rate of HCC in adults with HCV infection, the recurrence rate and progression of HCC in adults with HCV-related HCC. Final clinical outcomes include mortality and morbidity from HCV (including cirrhosis-associated complications, decompensation, liver transplantation, and survival) and harms of antiviral therapies (including influenza-like symptoms, hematologic effects, and psychiatric effects...).

HCV = hepatitis C virus; **HCC=** hepatocellular carcinoma; **KQ** = key question; **SVR** = sustained virologic response; **DAAs** = direct antiviral agents.

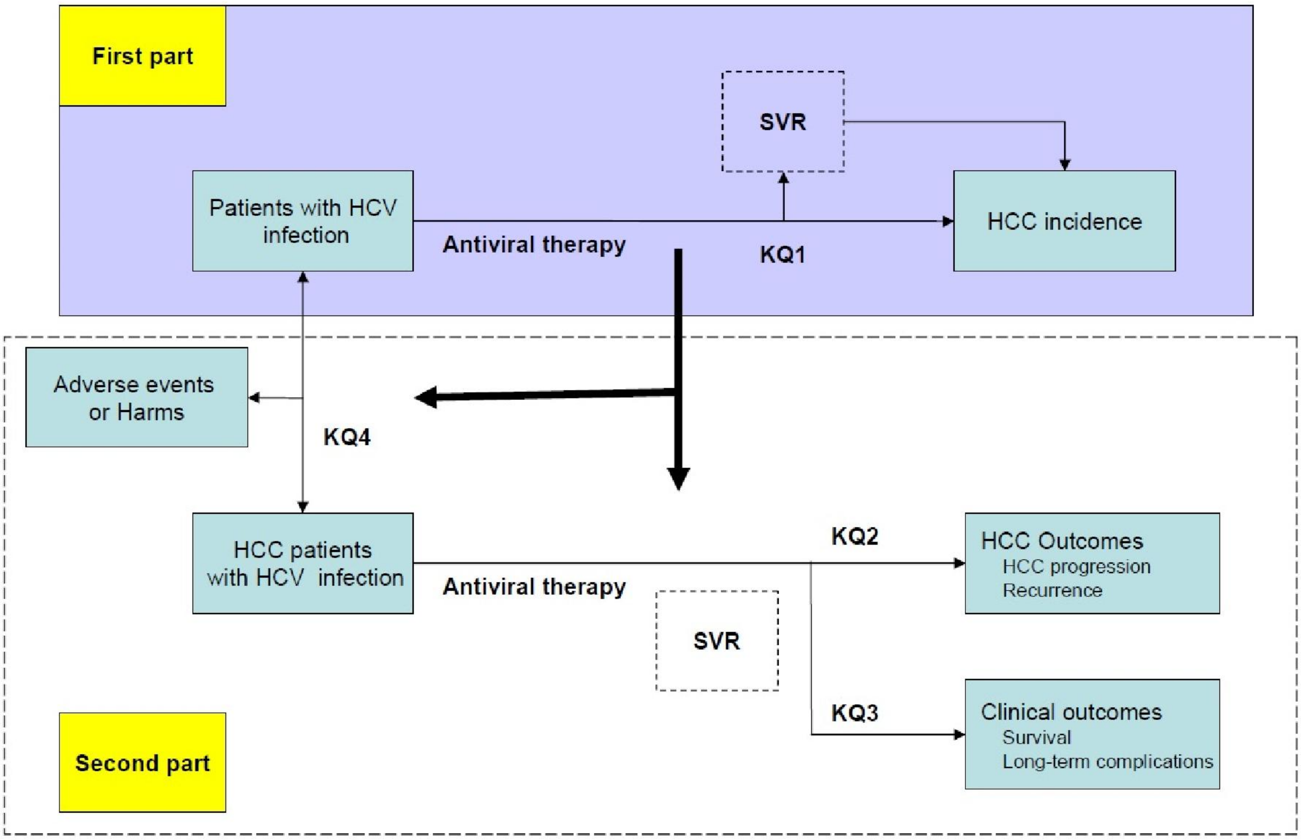
First part
SVR
Patients with HCV infection
Antiviral therapy
KQ1
HCC incidence
Adverse events or Harms
KQ4
HCC patients with HCV infection
Antiviral therapy
KQ2
HCC Outcomes
HCC progression
Recurrence
SVR
KQ3
Clinical outcomes
Survival
Long-term complications
Second part

Supplementary 2.

Review protocol of "Systemic Review: Impact of Interferon-based Therapy on HCV-related Hepatocellular Carcinoma"

Review objective

The 1st Part:

The objective is to examine the impact of antiviral therapy on the long-term HCC incidence of HCV patients.

Once a positive findings is shown in the first part, we proceed to the second part of this review.

The 2nd part:

The objective is to examine the impact of antiviral therapy on the long-term outcomes of HCV-related HCC patients.

Participants

The 1st Part:

Studies of participants diagnosed with HCV infection at the age of 18 years or above.

The 2nd part:

Studies of participants diagnosed with HCV-related HCC at the age of 18 years or above.

Interventions

Any intervention or combination of interventions given for the treatment of patients with chronic hepatitis C infection, including (but not restricted to) mono-therapy with interferon (IFN) or pegylated interferon (PEG-IFN) alfa; dual therapy with IFN plus ribavirin or PEG-IFN alfa plus ribavirin; and triple therapy with PEG-IFN (alfa-2a or -2b), ribavirin, and either telaprevir or boceprevir or newer direct antivial agents (DAAs). Studies using regimens with antiviral drugs not approved by the U.S. Food and Drug Administration for HCV infection are excluded.

Outcomes

The 1st Part:

The incidence rate of HCC.

The 2nd part:

Clinical outcomes were mortality, cirrhosis, hepatic decompensation, hepatocellular carcinoma, and need for transplantation.

HCC outcomes were the recurrence and progression rate of HCC.
Sustained virological response (SVR) was defined as the absence of detectable HCV RNA in the serum 6 months after the end of a course of therapy.
Biological response (BR) was defined as a decrease in serum ALT to within the normal range after treatment.
Harms included withdrawals due to adverse events, serious adverse events, neutropenia, anemia, psychological adverse events, influenza-like symptoms, and rash.

Study design

Randomised controlled trials (RCTs).
